# Synthesis and crystal structure of 1,3-di-*tert*-butyl-2-chloro-4,4-diphenyl-1,3,2λ^3^,4-di­aza­phospha­siletidine

**DOI:** 10.1107/S2056989019002627

**Published:** 2019-02-28

**Authors:** Dennis Mo, Walter Frank

**Affiliations:** a Institut für Anorganische Chemie und Strukturchemie, Lehrstuhl II: Material- und Strukturforschung, Heinrich-Heine-Universität Düsseldorf, Universitätsstrasse 1, D-40225 Düsseldorf, Germany

**Keywords:** di­aza­phosphasiletidine, four-membered heterocycle, crystal structure, long P—Cl bond

## Abstract

The chemical reaction of di­lithium *N*,*N*′-di(^*t*^but­yl)-*Si*,*Si*-di­phenyl­silanedi­amide and PCl_3_ yielded an off-white solid. Sublimation of the crude product under reduced pressure at elevated temperature gave colourless prismatic crystals of the title compound, C_20_H_28_ClN_2_PSi, which crystallizes in the non-centrosymmetric monoclinic space group *Cc.* The asymmetric unit of the crystal structure contains one mol­ecule and it is dominated by the central SiN_2_P four-membered ring, which is almost planar with a mean deviation of the atoms from the best plane of 0.014 Å. The angles between the plane defined by the silicon atom and the two nitro­gen atoms and the best planes of the *Si*-phenyl groups are 85.1 (2) and 77.4 (2)°, with the tilt of the phenyl rings in the opposite direction. Both *tert*-butyl groups suffer from a two-position rotational disorder with site occupancies of 0.752 (6)/0.248 (6) and 0.878 (9)/0.122 (9). The P—Cl bond [2.2078 (17) Å] is remarkably elongated compared to the P—Cl distance in PCl_3_ [2.034 Å; Galy & Enjalbert (1982). *J. Solid State Chem.* **44**, 1–23].

## Abstract 2

A new *Si*,*Si*-diphenyl-, *P*-chloro­functionalized di­aza­phosphasiletidine has been synthesized. The crystal structure of the highly moisture-sensitive four-membered heterocyclic mol­ecular compound, which has a remarkably elongated P—Cl bond, is described and compared with related compounds and their arrangement of mol­ecules in the solid state.

## Chemical context   

Di­aza­phosphasiletidines are heterocyclic compounds that contain an SiN_2_P four-membered ring as the central building block. The first synthesis was described in the year 1963 (Fink, 1963 ▸) and compounds of the class have attracted considerable attention in phospho­rus chemistry (*e.g.* Scherer *et al.*, 1982 ▸; Veith *et al.*, 1988 ▸; Frank *et al.*, 1996 ▸; Mo *et al.*, 2018 ▸). The *P*-chloro­substituted di­aza­phosphasiletidines are well known members of this class and syntheses of such compounds have been described in the literature over a couple of decades (Klingebiel *et al.*, 1976 ▸; Veith *et al.*, 1988 ▸; Eichhorn & Nöth, 2000 ▸). They have found widespread use as reagents for reactions based on the *P*-chloro­functionalization. Our research group, for instance, has shown that they play a crucial role in the preparation of di­spiro­cyclic tetra­phosphetes (Frank *et al.*, 1996 ▸; Breuers *et al.*, 2015 ▸) and di­aza­phosphasiletidine adducts with *P*-coordination (Veith *et al.*, 1988 ▸; Gün *et al.*, 2017 ▸). However, due to their high moisture sensitivity, the structural characterization of such *P*-chloro­derivatives by X-ray diffraction remains a challenge. There are only two reports on the crystal structure of *P*-chloro­substituted di­aza­phosphasiletidines of type Me_2_Si(N*R*)_2_PCl, namely 2-chloro-1,3-bis­(2,4,6-tri­methyl­phen­yl)-4,4-dimethyl-1,3,2λ^3^,4-di­aza­phosphasiletidine (**A**; Breuers & Frank, 2016 ▸) and 1,3-di-*tert*-butyl-2-chloro-4,4-dimethyl-1,3,2λ^3^,4-di­aza­phosphasiletidine (**B**; Gün *et al.*, 2017 ▸), and there is only one report on a structure of type Ph_2_Si(N*R*)_2_PCl, namely 2-chloro-1,3-di-*tert*-pentyl-4,4-diphenyl-1,3,2λ^3^,4-di­aza­phosphasiletidine (**C**; Mo *et al.*, 2018 ▸). Crystals of the first structurally characterized chloro-substituted di­aza­phosphasiletidine **A** contained approximately 12% of a second compound, namely 2-chloro-1,3-bis­(2,4,6-tri­methyl­phen­yl)-4-chloro-4-methyl-1,3,2λ^3^,4-di­aza­phosphasiletidine. With respect to this impurity, an *Si*,*Si*-diphenyl-substituted di­aza­phosphasiletidine (**C**) has successfully been introduced to preparative chemistry to avoid problems related to the content of *Si*,*P*-bis­(chloro)­functionalized species present in samples of the *Si*,*Si*-di­methyl ­derivative. However, the crystal-structure determination of **C** suffered from severe disorder. All the aspects mentioned before persuaded us to focus on preparation of single crystals of the title compound suitable for structure determination. After extensive attempts, we were finally able to grow single crystals by slow sublimation *in vacuo* and confirmed its composition and its structure *via* X-ray diffraction.
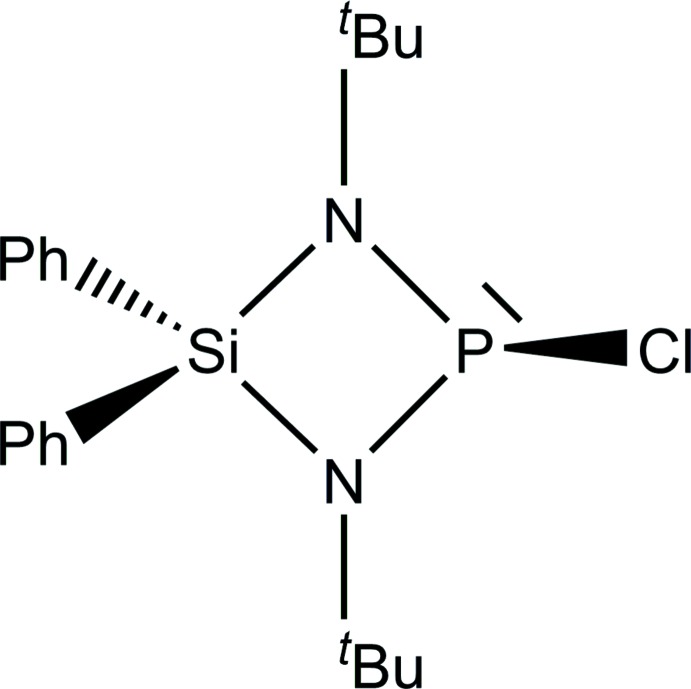


## Structural commentary   

**Figure 1:**
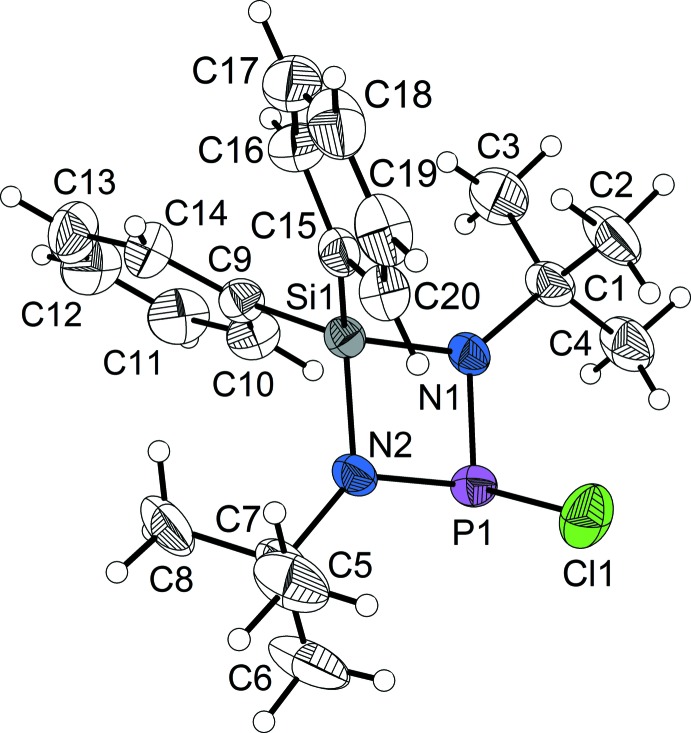
The mol­ecular structure of the title compound, with atom labels and 50% probability displacement ellipsoids for non-H atoms.

The asymmetric unit of the title compound contains one mol­ecule (Fig. 1 ▸). The central feature of this di­aza­phosphasiletidine mol­ecule, the SiN_2_P four-membered ring, is almost planar. The nitro­gen atoms exhibit a trigonal–planar coordination sphere [sums of bond angles 359.9° (N1) and 359.4° (N2)]. The phospho­rus and silicon atoms bear the main ring strain [N1—Si1—N2 = 82.08 (19)° and N1—P1—N2 = 85.4 (2)°]. The Si–N bond lengths [Si1—N1 = 1.736 (4) Å and Si1—N2 =1.749 (4) Å] exceed the expected length of an Si—N single bond [1.724 (4) Å; Brown *et al.*, 1985 ▸] but correspond to those in directly related cyclo­silaza­nes (Breuers *et al.*, 2016 ▸; Gün *et al.*, 2017 ▸; Clegg *et al.*, 1981 ▸, 1984 ▸; Shah *et al.*, 1996 ▸; Anagho *et al.*, 2005 ▸). In contrast, the P—N distances are shorter [P1—N1 = 1.689 (4) Å and P1—N2 = 1.684 (4) Å] than reported for a typical single bond [1.704 (9) Å; Brown *et al.*, 1985 ▸], but they also correspond to those in **A**–**C**. The P—Cl bond of the title compound is remarkably elongated [P1—Cl1 = 2.2078 (17) Å] compared to the P—Cl distance in PCl_3_ (2.034 Å; Galy *et al.*, 1982 ▸) and exceeds the sum of the covalence radii (Hollemann *et al.*, 2007 ▸). A comparison of the average Si—N, P—N and P—Cl distances in the title compound and the analogous distances of in the previously published *P*-chloro-substituted di­aza­phosphasiletidines **A**–**C** gives no evidence of substitution effects except for the P—Cl distance in **B** [2.2498 (6) Å, due to dimerization]: Si—N = 1.743 (4) Å average (in the title compound) *vs* 1.7441 (17) Å in **A**, 1.7474 (14) Å in **B** and 1.7406 (15) Å in **C** (average values); P—N = 1.687 (4) Å *vs* 1.6856 (17) Å (**A**), 1.6815 (14) Å (**B**), 1.6910 (16) Å (**C**); P—Cl 2.2078 (17) Å *vs* 2.1813 (7) Å (**A**), 2.2498 (6) Å (**B**) (dimerization), 2.1965 (17) Å (**C**). The *tert*-butyl groups in the title compound are rotationally disordered (see *Refinement*).

## Supra­molecular features   

**Figure 2:**
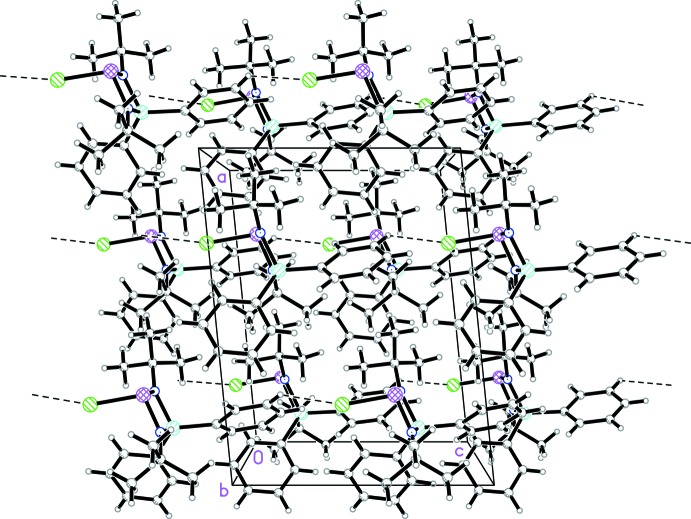
Packing of the mol­ecules of the title compound in the solid state. The closest contact of the Cl atom to neighbouring mol­ecules is indicated by dashed lines.

Fig. 2 ▸ shows the arrangement of mol­ecules in the non-centrosymmetric solid of the title compound. Taking into account its absolute structure, in the crystal under investigation the P—Cl bond vectors are oriented approximately parallel to the *c* axis, but point in the opposite direction. The nearest inter­molecular contact is between the Cl atom and the *meta-*H atom of one of the Si-bonded phenyl groups of a neighbouring mol­ecule (symmetry code: *x*, *y*, –*z*). In the figure, this contact is indicated by dashed lines. However, the geometric features of this contact [C⋯Cl 3.677 (6); C—H 0.95; H⋯Cl 2.90 Å; C—H⋯Cl 139°] indicate that if at all, it is a borderline case of a directed bonding inter­action.

## Database survey   

A search in the Cambridge Structural Database (Version 5.40, November 2018; Groom *et al.*, 2016 ▸) for di­aza­phosphasiletidines in general yielded 143 hits. However, only three of these structures contain an Si,Si-diphenyl fragment instead of the common Si,Si-dimethyl fragment. On the other hand, only seven of the aforementioned 143 structures exhibit *P*-chloro­functionalization. Of these, BADLUO (Nieger *et al.*, 2002 ▸) is a λ^5^ *P*-chloro­(imino)­phospho­rane, VUHTOJ (Holt­hausen & Weigand, 2016 ▸) contains a complex *N*,*N*′-tri­methyl­silyl-Si-di­spiro­cyclic cation incorporating a tricylic P_5_ fragment. ILEKER is the *N*,*N*′-dimesityl derivative **A**, mentioned above (Breuers & Frank, 2016 ▸). DEXTOS is the *Si*,*Si*-dimethyl derivative **B**, mentioned above, accompanied in Gün *et al.* (2017 ▸) by its BCl_3_ adduct DEXTUY and its W(CO)_5_ complex DEXVAG. The structure of the only *Si*,*Si*-diphenyl-*P*-chloro derivative (**C**), 2-chloro-1,3-*bis*(2-methyl­butan-2-yl)-4,4-diphenyl-1,3,2λ^3^,4-di­aza­phosphasiletidine (YETCAE; Mo *et al.*, 2018 ▸) suffers heavily from a combination of several types of disorder of the *N*,*N*′-alkyl substituents.

In compound **B**, mol­ecules are connected *via* very weak P—Cl bridging bonds, which leads to a weak state of dimerization. Generally, the strength of association of mol­ecules *via E*—Cl bridging bonds increases from P to Bi in related di­aza­sileditines of type Me_2_Si(N*R*)_2_ *E*Cl. Me_2_Si(N^*t*^Bu)_2_AsCl contains dimers and in the anti­mony and the bis­muth analogues the mol­ecules are connected into chains *via* bridging Cl atoms (Veith & Bertsch, 1988 ▸; Veith *et al.*, 1988 ▸). In contrast, the solid-state structures of the title compound, **A**, **C**, Ph_2_Si(N^*t*^Bu)_2_AsCl (Belter, 2016 ▸) and Me_2_Si(NDipp)_2_SbCl (Ma *et al.*, 2013 ▸) do not exhibit inter­molecular *E*⋯Cl inter­actions and consist of isolated mol­ecules.

## Synthesis and crystallization   

**Figure 3:**
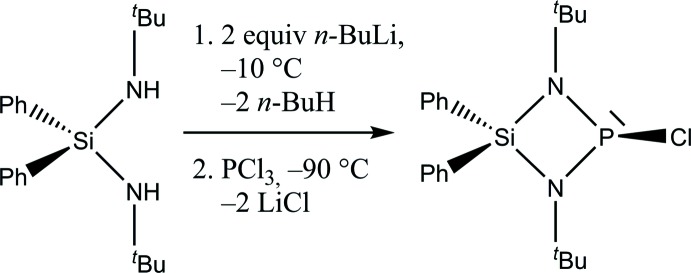
Reaction scheme for the preparation of the title compound.

The title compound was prepared (Fig. 3 ▸) according to generally known procedures under an argon atmosphere in oven-dried glassware using Schlenk techniques, modifying a published protocol (Eichhorn & Nöth, 2000 ▸). 5.5 g (16.8 mmol) of *N*,*N*′-di(^*t*^but­yl)-*Si*,*Si*-di­phenyl­silanedi­amine were dissolved in 60 ml *n*-pentane. 13.6 ml of a *n*-butyl­lithium solution (*c* = 2.5 mol/l in *n*-hexane, 16.8 mmol) were added at 263 K. The reaction mixture was stirred for 24 h at room temperature. Cooling to 178 K and addition of 1.5 ml (16.8 mmol) PCl_3_ yielded an off-white suspension. This was stirred for 3 h. After filtration and removal of the solvent under reduced pressure, the crude product was obtained as an off-white solid. Sublimation at 333 K under reduced pressure yielded colourless crystals within a couple of hours (77% yield based on PCl_3_). **^1^H NMR** (300 MHz, CDCl_3_, 298 K): δ (p.p.m.) 1.17 (*d*, ^4^ *J* (P,H) = 0.9 Hz, 18H,C(C**H**_3_)_3_), 7.48 (*m*, 6H, *m*-, *p*-C**H**), 7.86 (*m*, 2H,*o*-C**H**), 8.08 (*m*, 2H, *o*-C**H**). **^13^C{^1^H} NMR** (75 MHz, CDCl_3_, 298 K): δ(p.p.m.) 32.9 [*d*, ^3^ *J*(P,*C*) = 7.1 Hz, 6 C, C(**C**H_3_)_3_], 52.6 [*d*, ^2^ *J*(P,*C*) = 7.9 Hz, 2 C, **C**(CH_3_)_3_], 128.3–136.3 (12 C, Ar-**C**). **^31^P{^1^H} NMR** (121 MHz, CDCl_3_, 298 K): δ (p.p.m.) 214.4 (*s*) EI–MS spectra were obtained using a Finnigan TSQ 7000 instrument. EI–MS: *m*/z (%) 390 (11) [*M* ^+^], 375 (100) [*M* ^+^—C(CH_3_)_3_]. IR spectra were measured using a Bio-Rad Excalibur FTS 3500 FT–IR spectrometer with ATR-unit, 4000–560 cm^−1^: 3070(*w*), 3050(*w*), 3026(*sh*), 3014(*sh*), 2956(*vs*), 2927(*s*), 2903(*sh*), 2868(*m*), 1964(*vw*), 1903(*vw*), 1827(*vw*), 1774(*vw*), 1588(*w*), 1429(*s*), 1305(*vw*), 1207(*s*), 1113(*s*), 1102(*sh*), 1055(*s*), 1042(*sh*), 889(*vs*), 820(*w*), 755(*sh*), 739(*s*), 696(*s*). Analysis calculated for C_20_H_28_ClN_2_PSi (326.56 g mol^−1^): C 61.44, H 7.22, N 7.17; found C 61.10, H 7.56, N 7.08, m.p.: 393.5 K.

## Refinement   

Crystal data, data collection and structure refinement details are summarized in Table 1 ▸. Positions of the majority of the hydrogen atoms were identified *via* subsequent Fourier syntheses. In the refinement, a riding model was applied using idealized C—H bond lengths (0.95–0.98 Å) as well as H—C—H and C—C—H angles. In addition, the H atoms of the CH_3_ groups were allowed to rotate around the neighboring C—C bonds. The *U*_iso_ values were set to 1.5*U*_eq_(C_meth­yl_) and 1.2*U*_eq_(C_ar_). To account for residual electron density in the regions of the two *tert*-butyl groups and for elongated anisotropic displacement ellipsoids of several carbon atoms that did not appear to be physically meaningful, a two-position disorder for each *tert*-butyl group was introduced with partial occupation sites for all carbon atoms but the tertiary ones C1 and C5 [occupancy ratio 0.752 (6):0.248 (6) ratio (group containing C1) and 0.878 (9):0.122 (9) ratio (C5); in Figs. 1 ▸ and 2 ▸ disorder is omitted for clarity]. Appropriate same distance and anisotropic displacement restraints and some equivalent anisotropic displacement parameters had to be applied to stabilize the geometry of the minor occupancy parts of the partial occupation site models. The correct absolute structure of the non-centrosymmetric structural model is confirmed by the Flack parameter (Table 1 ▸).

## Supplementary Material

Crystal structure: contains datablock(s) I, global. DOI: 10.1107/S2056989019002627/pk2614sup1.cif

Structure factors: contains datablock(s) I. DOI: 10.1107/S2056989019002627/pk2614Isup2.hkl

CCDC reference: 1898427

Additional supporting information:  crystallographic information; 3D view; checkCIF report

## Figures and Tables

**Table 1:** Experimental details

Crystal data	
Chemical formula	C_20_H_28_ClN_2_PSi
*M*_r_	390.95
Crystal system, space group	Monoclinic, *C* *c*
Temperature (K)	173
*a*, *b*, *c* (Å)	13.4004 (7), 15.6272 (6), 10.3817 (5)
β (°)	95.739 (4)
*V* (Å^3^)	2163.14 (18)
*Z*	4
Radiation type	Mo *K*α
μ (mm^−1^)	0.31
Crystal size (mm)	0.44 × 0.38 × 0.21

Data collection	
Diffractometer	Stoe IPDS
Absorption correction	Multi-scan (*SHELXTL*; Sheldrick, 2008 ▸)
*T*_min_, *T*_max_	0.688, 0.875
No. of measured, independent and observed [*I* > 2σ(*I*)] reflections	11994, 5765, 4920
*R*_int_	0.064
(sin θ/λ)_max_ (Å^−1^)	0.684

Refinement	
*R*[*F* ^2^ > 2σ(*F* ^2^)], *wR*(*F* ^2^), *S*	0.063, 0.104, 1.50
No. of reflections	5765
No. of parameters	255
No. of restraints	32
H-atom treatment	H-atom parameters constrained
Δρ_max_, Δρ_min_ (e Å^−3^)	0.26, −0.27
Absolute structure	Flack *x* determined using 1837 quotients [(*I* ^+^)−(*I* ^−^)]/[(*I* ^+^)+(*I* ^−^)] (Parsons *et al.*, 2013 ▸)
Absolute structure parameter	0.08 (8)

Computer programs: *X-AREA* (Stoe & Cie, 2009 ▸), *SHELXT* (Sheldrick, 2015*a* ▸), *DIAMOND* (Brandenburg, 2016 ▸), *SHELXL2014/7* (Sheldrick, 2015*b* ▸) and *publCIF* (Westrip, 2010 ▸).

## supplementary crystallographic information

### Crystal data

**Table:**

C_20_H_28_ClN_2_PSi	*D*_x_ = 1.201 Mg m^−^^3^
*M**_r_* = 390.95	Melting point: 393.5 K
Monoclinic, *C**c*	Mo *K*α radiation, λ = 0.71073 Å
*a* = 13.4004 (7) Å	Cell parameters from 12536 reflections
*b* = 15.6272 (6) Å	θ = 2.6–29.7°
*c* = 10.3817 (5) Å	µ = 0.31 mm^−^^1^
β = 95.739 (4)°	*T* = 173 K
*V* = 2163.14 (18) Å^3^	Prismatic, colourless
*Z* = 4	0.44 × 0.38 × 0.21 mm
*F*(000) = 832	

### Data collection

**Table:**

Stoe IPDS diffractometer	5765 independent reflections
Radiation source: sealed tube	4920 reflections with *I* > 2σ(*I*)
Graphite monochromator	*R*_int_ = 0.064
ω scans	θ_max_ = 29.1°, θ_min_ = 2.6°
Absorption correction: multi-scan (SHELXTL; Sheldrick, 2008)	*h* = −18→18
*T*_min_ = 0.688, *T*_max_ = 0.875	*k* = −20→21
11994 measured reflections	*l* = −14→14

### Refinement

**Table:**

Refinement on *F*^2^	Secondary atom site location: difference Fourier map
Least-squares matrix: full	Hydrogen site location: inferred from neighbouring sites
*R*[*F*^2^ > 2σ(*F*^2^)] = 0.063	H-atom parameters constrained
*wR*(*F*^2^) = 0.104	*w* = 1/[σ^2^(*F*_o_^2^) + (0.008*P*)^2^ + 1.4244*P*] where *P* = (*F*_o_^2^ + 2*F*_c_^2^)/3
*S* = 1.50	(Δ/σ)_max_ = 0.001
5765 reflections	Δρ_max_ = 0.26 e Å^−^^3^
255 parameters	Δρ_min_ = −0.27 e Å^−^^3^
32 restraints	Absolute structure: Flack *x* determined using 1837 quotients [(*I*^+^)-(*I*^-^)]/[(*I*^+^)+(*I*^-^)] (Parsons *et al.*, 2013)
Primary atom site location: structure-invariant direct methods	Absolute structure parameter: 0.08 (8)

### Special details

**Table:**

Geometry. All esds (except the esd in the dihedral angle between two l.s. planes) are estimated using the full covariance matrix. The cell esds are taken into account individually in the estimation of esds in distances, angles and torsion angles; correlations between esds in cell parameters are only used when they are defined by crystal symmetry. An approximate (isotropic) treatment of cell esds is used for estimating esds involving l.s. planes.

### Fractional atomic coordinates and isotropic or equivalent isotropic displacement parameters (Å^2^)

**Table:**

	*x*	*y*	*z*	*U*_iso_*/*U*_eq_	Occ. (<1)
Cl1	0.72280 (12)	0.34044 (10)	0.44721 (13)	0.0555 (4)	
P1	0.75140 (9)	0.32990 (8)	0.65977 (11)	0.0340 (3)	
Si1	0.63594 (9)	0.21721 (8)	0.74331 (11)	0.0292 (3)	
N1	0.6359 (3)	0.3262 (2)	0.7115 (4)	0.0327 (9)	
N2	0.7534 (3)	0.2234 (3)	0.6842 (4)	0.0347 (9)	
C1	0.5626 (4)	0.3962 (3)	0.7149 (5)	0.0403 (11)	
C2	0.4879 (6)	0.3880 (5)	0.5905 (9)	0.059 (2)	0.752 (6)
H21	0.4348	0.4309	0.5925	0.089*	0.752 (6)
H22	0.4582	0.3306	0.5867	0.089*	0.752 (6)
H23	0.5238	0.3972	0.5139	0.089*	0.752 (6)
C3	0.5049 (7)	0.3845 (6)	0.8317 (10)	0.073 (3)	0.752 (6)
H31	0.4508	0.4268	0.8292	0.110*	0.752 (6)
H32	0.5503	0.3924	0.9109	0.110*	0.752 (6)
H33	0.4763	0.3268	0.8307	0.110*	0.752 (6)
C4	0.6140 (6)	0.4825 (4)	0.7091 (9)	0.0531 (19)	0.752 (6)
H41	0.5638	0.5282	0.7083	0.080*	0.752 (6)
H42	0.6484	0.4858	0.6303	0.080*	0.752 (6)
H43	0.6630	0.4892	0.7851	0.080*	0.752 (6)
C2A	0.5825 (18)	0.4340 (15)	0.8546 (19)	0.059 (2)	0.248 (6)
H24	0.5403	0.4847	0.8622	0.089*	0.248 (6)
H25	0.6533	0.4502	0.8713	0.089*	0.248 (6)
H26	0.5664	0.3909	0.9179	0.089*	0.248 (6)
C3A	0.579 (2)	0.4643 (16)	0.619 (3)	0.073 (3)	0.248 (6)
H34	0.5288	0.5095	0.6240	0.110*	0.248 (6)
H35	0.5728	0.4397	0.5319	0.110*	0.248 (6)
H36	0.6463	0.4885	0.6386	0.110*	0.248 (6)
C4A	0.4558 (13)	0.3610 (13)	0.712 (3)	0.0531 (19)	0.248 (6)
H44	0.4086	0.4085	0.7168	0.080*	0.248 (6)
H45	0.4514	0.3224	0.7853	0.080*	0.248 (6)
H46	0.4391	0.3295	0.6307	0.080*	0.248 (6)
C5	0.8384 (4)	0.1638 (4)	0.6749 (5)	0.0464 (12)	
C6	0.9360 (5)	0.2115 (6)	0.6854 (13)	0.084 (3)	0.878 (9)
H61	0.9906	0.1718	0.6721	0.126*	0.878 (9)
H62	0.9480	0.2374	0.7716	0.126*	0.878 (9)
H63	0.9332	0.2566	0.6194	0.126*	0.878 (9)
C7	0.8229 (6)	0.1177 (5)	0.5448 (8)	0.071 (2)	0.878 (9)
H71	0.8780	0.0773	0.5377	0.106*	0.878 (9)
H72	0.8215	0.1597	0.4745	0.106*	0.878 (9)
H73	0.7591	0.0865	0.5386	0.106*	0.878 (9)
C8	0.8350 (6)	0.0952 (6)	0.7785 (8)	0.075 (3)	0.878 (9)
H81	0.8821	0.0493	0.7628	0.112*	0.878 (9)
H82	0.7670	0.0718	0.7755	0.112*	0.878 (9)
H83	0.8539	0.1204	0.8639	0.112*	0.878 (9)
C6A	0.799 (4)	0.075 (2)	0.647 (9)	0.084 (3)	0.122 (9)
H64	0.8552	0.0353	0.6409	0.126*	0.122 (9)
H65	0.7599	0.0560	0.7170	0.126*	0.122 (9)
H66	0.7558	0.0744	0.5649	0.126*	0.122 (9)
C7A	0.890 (4)	0.160 (4)	0.814 (3)	0.071 (2)	0.122 (9)
H74	0.9482	0.1210	0.8164	0.106*	0.122 (9)
H75	0.9131	0.2169	0.8416	0.106*	0.122 (9)
H76	0.8430	0.1381	0.8722	0.106*	0.122 (9)
C8A	0.915 (4)	0.216 (4)	0.608 (7)	0.075 (3)	0.122 (9)
H84	0.9742	0.1814	0.5984	0.112*	0.122 (9)
H85	0.8848	0.2350	0.5230	0.112*	0.122 (9)
H86	0.9345	0.2668	0.6612	0.112*	0.122 (9)
C9	0.6452 (4)	0.1891 (3)	0.9186 (4)	0.0350 (10)	
C10	0.6963 (4)	0.2448 (4)	1.0070 (5)	0.0455 (12)	
H101	0.7210	0.2975	0.9776	0.055*	
C11	0.7117 (4)	0.2239 (4)	1.1385 (5)	0.0547 (14)	
H111	0.7460	0.2627	1.1979	0.066*	
C12	0.6774 (5)	0.1475 (5)	1.1817 (5)	0.0598 (16)	
H121	0.6877	0.1338	1.2712	0.072*	
C13	0.6286 (5)	0.0909 (4)	1.0973 (5)	0.0596 (16)	
H131	0.6052	0.0379	1.1276	0.072*	
C14	0.6134 (4)	0.1115 (3)	0.9657 (5)	0.0451 (12)	
H141	0.5805	0.0715	0.9070	0.054*	
C15	0.5370 (3)	0.1539 (3)	0.6457 (4)	0.0350 (10)	
C16	0.4537 (4)	0.1194 (4)	0.6972 (6)	0.0493 (13)	
H161	0.4452	0.1282	0.7860	0.059*	
C17	0.3831 (4)	0.0724 (4)	0.6203 (7)	0.0622 (17)	
H171	0.3274	0.0483	0.6571	0.075*	
C18	0.3932 (5)	0.0604 (4)	0.4901 (7)	0.0632 (18)	
H181	0.3452	0.0274	0.4380	0.076*	
C19	0.4732 (5)	0.0966 (4)	0.4362 (6)	0.0534 (14)	
H191	0.4796	0.0899	0.3464	0.064*	
C20	0.5442 (4)	0.1429 (3)	0.5136 (5)	0.0417 (11)	
H201	0.5990	0.1677	0.4758	0.050*	

### Atomic displacement parameters (Å^2^)

**Table:**

	*U*^11^	*U*^22^	*U*^33^	*U*^12^	*U*^13^	*U*^23^
Cl1	0.0735 (10)	0.0566 (9)	0.0376 (6)	0.0008 (8)	0.0126 (6)	0.0061 (6)
P1	0.0318 (6)	0.0347 (6)	0.0360 (6)	−0.0002 (6)	0.0057 (5)	−0.0022 (5)
Si1	0.0298 (6)	0.0277 (6)	0.0301 (6)	0.0026 (5)	0.0030 (4)	−0.0025 (5)
N1	0.032 (2)	0.0295 (19)	0.038 (2)	0.0074 (17)	0.0075 (17)	−0.0009 (15)
N2	0.031 (2)	0.039 (2)	0.034 (2)	0.0092 (18)	0.0066 (17)	0.0012 (17)
C1	0.035 (3)	0.032 (2)	0.055 (3)	0.008 (2)	0.007 (2)	−0.005 (2)
C2	0.041 (4)	0.045 (4)	0.088 (6)	0.015 (3)	−0.013 (4)	−0.005 (4)
C3	0.067 (6)	0.063 (5)	0.098 (7)	0.026 (5)	0.041 (5)	0.004 (5)
C4	0.046 (4)	0.035 (3)	0.076 (5)	0.008 (3)	−0.001 (4)	−0.007 (3)
C2A	0.041 (4)	0.045 (4)	0.088 (6)	0.015 (3)	−0.013 (4)	−0.005 (4)
C3A	0.067 (6)	0.063 (5)	0.098 (7)	0.026 (5)	0.041 (5)	0.004 (5)
C4A	0.046 (4)	0.035 (3)	0.076 (5)	0.008 (3)	−0.001 (4)	−0.007 (3)
C5	0.036 (3)	0.046 (3)	0.058 (3)	0.016 (2)	0.008 (2)	−0.003 (3)
C6	0.030 (3)	0.070 (5)	0.151 (9)	0.010 (4)	0.002 (4)	−0.032 (6)
C7	0.061 (5)	0.067 (5)	0.087 (6)	0.024 (4)	0.020 (4)	−0.025 (4)
C8	0.062 (5)	0.072 (5)	0.094 (6)	0.043 (4)	0.024 (4)	0.026 (5)
C6A	0.030 (3)	0.070 (5)	0.151 (9)	0.010 (4)	0.002 (4)	−0.032 (6)
C7A	0.061 (5)	0.067 (5)	0.087 (6)	0.024 (4)	0.020 (4)	−0.025 (4)
C8A	0.062 (5)	0.072 (5)	0.094 (6)	0.043 (4)	0.024 (4)	0.026 (5)
C9	0.036 (2)	0.038 (2)	0.031 (2)	0.0016 (19)	0.0042 (18)	−0.0014 (19)
C10	0.046 (3)	0.050 (3)	0.040 (3)	−0.003 (2)	0.006 (2)	−0.004 (2)
C11	0.055 (3)	0.074 (4)	0.034 (3)	−0.007 (3)	−0.003 (2)	−0.008 (3)
C12	0.061 (4)	0.090 (5)	0.028 (2)	−0.002 (4)	0.002 (2)	0.008 (3)
C13	0.071 (4)	0.063 (4)	0.045 (3)	0.004 (3)	0.009 (3)	0.016 (3)
C14	0.053 (3)	0.041 (3)	0.041 (3)	−0.005 (2)	0.003 (2)	0.004 (2)
C15	0.036 (2)	0.029 (2)	0.038 (2)	0.0033 (19)	−0.0021 (18)	0.0018 (19)
C16	0.038 (3)	0.055 (3)	0.054 (3)	−0.007 (3)	0.002 (2)	0.003 (3)
C17	0.042 (3)	0.064 (4)	0.077 (4)	−0.012 (3)	−0.007 (3)	0.016 (3)
C18	0.054 (4)	0.051 (3)	0.078 (4)	−0.010 (3)	−0.028 (3)	0.002 (3)
C19	0.064 (4)	0.049 (3)	0.043 (3)	0.005 (3)	−0.014 (3)	−0.009 (2)
C20	0.046 (3)	0.038 (3)	0.040 (2)	0.003 (2)	−0.001 (2)	−0.003 (2)

### Geometric parameters (Å, º)

**Table:**

Cl1—P1	2.2078 (17)	C6—H62	0.9800
P1—N2	1.684 (4)	C6—H63	0.9800
P1—N1	1.689 (4)	C7—H71	0.9800
Si1—N1	1.736 (4)	C7—H72	0.9800
Si1—N2	1.749 (4)	C7—H73	0.9800
Si1—C9	1.864 (5)	C8—H81	0.9800
Si1—C15	1.869 (5)	C8—H82	0.9800
N1—C1	1.473 (6)	C8—H83	0.9800
N2—C5	1.481 (6)	C6A—H64	0.9800
C1—C3A	1.487 (18)	C6A—H65	0.9800
C1—C3	1.512 (9)	C6A—H66	0.9800
C1—C4	1.518 (8)	C7A—H74	0.9800
C1—C4A	1.530 (17)	C7A—H75	0.9800
C1—C2	1.559 (8)	C7A—H76	0.9800
C1—C2A	1.564 (17)	C8A—H84	0.9800
C2—H21	0.9800	C8A—H85	0.9800
C2—H22	0.9800	C8A—H86	0.9800
C2—H23	0.9800	C9—C14	1.390 (7)
C3—H31	0.9800	C9—C10	1.394 (7)
C3—H32	0.9800	C10—C11	1.398 (7)
C3—H33	0.9800	C10—H101	0.9500
C4—H41	0.9800	C11—C12	1.372 (9)
C4—H42	0.9800	C11—H111	0.9500
C4—H43	0.9800	C12—C13	1.365 (9)
C2A—H24	0.9800	C12—H121	0.9500
C2A—H25	0.9800	C13—C14	1.399 (7)
C2A—H26	0.9800	C13—H131	0.9500
C3A—H34	0.9800	C14—H141	0.9500
C3A—H35	0.9800	C15—C16	1.394 (7)
C3A—H36	0.9800	C15—C20	1.395 (6)
C4A—H44	0.9800	C16—C17	1.386 (8)
C4A—H45	0.9800	C16—H161	0.9500
C4A—H46	0.9800	C17—C18	1.385 (9)
C5—C6	1.500 (9)	C17—H171	0.9500
C5—C6A	1.51 (2)	C18—C19	1.379 (9)
C5—C8	1.523 (9)	C18—H181	0.9500
C5—C7	1.526 (8)	C19—C20	1.386 (7)
C5—C8A	1.53 (2)	C19—H191	0.9500
C5—C7A	1.54 (2)	C20—H201	0.9500
C6—H61	0.9800		
			
N2—P1—N1	85.4 (2)	C8A—C5—C7A	101 (4)
N2—P1—Cl1	102.87 (15)	C5—C6—H61	109.5
N1—P1—Cl1	104.31 (15)	C5—C6—H62	109.5
N1—Si1—N2	82.08 (19)	H61—C6—H62	109.5
N1—Si1—C9	114.6 (2)	C5—C6—H63	109.5
N2—Si1—C9	112.4 (2)	H61—C6—H63	109.5
N1—Si1—C15	115.4 (2)	H62—C6—H63	109.5
N2—Si1—C15	117.0 (2)	C5—C7—H71	109.5
C9—Si1—C15	112.3 (2)	C5—C7—H72	109.5
C1—N1—P1	128.1 (3)	H71—C7—H72	109.5
C1—N1—Si1	135.4 (3)	C5—C7—H73	109.5
P1—N1—Si1	96.37 (19)	H71—C7—H73	109.5
C5—N2—P1	127.8 (4)	H72—C7—H73	109.5
C5—N2—Si1	135.5 (3)	C5—C8—H81	109.5
P1—N2—Si1	96.1 (2)	C5—C8—H82	109.5
N1—C1—C3A	111.8 (10)	H81—C8—H82	109.5
N1—C1—C3	109.0 (5)	C5—C8—H83	109.5
N1—C1—C4	110.6 (4)	H81—C8—H83	109.5
C3—C1—C4	114.2 (6)	H82—C8—H83	109.5
N1—C1—C4A	110.9 (9)	C5—C6A—H64	109.5
C3A—C1—C4A	116.3 (17)	C5—C6A—H65	109.5
N1—C1—C2	107.0 (4)	H64—C6A—H65	109.5
C3—C1—C2	108.5 (6)	C5—C6A—H66	109.5
C4—C1—C2	107.3 (6)	H64—C6A—H66	109.5
N1—C1—C2A	104.5 (9)	H65—C6A—H66	109.5
C3A—C1—C2A	109.1 (17)	C5—C7A—H74	109.5
C4A—C1—C2A	103.2 (15)	C5—C7A—H75	109.5
C1—C2—H21	109.5	H74—C7A—H75	109.5
C1—C2—H22	109.5	C5—C7A—H76	109.5
H21—C2—H22	109.5	H74—C7A—H76	109.5
C1—C2—H23	109.5	H75—C7A—H76	109.5
H21—C2—H23	109.5	C5—C8A—H84	109.5
H22—C2—H23	109.5	C5—C8A—H85	109.5
C1—C3—H31	109.5	H84—C8A—H85	109.5
C1—C3—H32	109.5	C5—C8A—H86	109.5
H31—C3—H32	109.5	H84—C8A—H86	109.5
C1—C3—H33	109.5	H85—C8A—H86	109.5
H31—C3—H33	109.5	C14—C9—C10	117.4 (4)
H32—C3—H33	109.5	C14—C9—Si1	123.8 (4)
C1—C4—H41	109.5	C10—C9—Si1	118.5 (4)
C1—C4—H42	109.5	C9—C10—C11	120.8 (5)
H41—C4—H42	109.5	C9—C10—H101	119.6
C1—C4—H43	109.5	C11—C10—H101	119.6
H41—C4—H43	109.5	C12—C11—C10	120.1 (5)
H42—C4—H43	109.5	C12—C11—H111	120.0
C1—C2A—H24	109.5	C10—C11—H111	120.0
C1—C2A—H25	109.5	C13—C12—C11	120.6 (5)
H24—C2A—H25	109.5	C13—C12—H121	119.7
C1—C2A—H26	109.5	C11—C12—H121	119.7
H24—C2A—H26	109.5	C12—C13—C14	119.4 (6)
H25—C2A—H26	109.5	C12—C13—H131	120.3
C1—C3A—H34	109.5	C14—C13—H131	120.3
C1—C3A—H35	109.5	C9—C14—C13	121.7 (5)
H34—C3A—H35	109.5	C9—C14—H141	119.2
C1—C3A—H36	109.5	C13—C14—H141	119.2
H34—C3A—H36	109.5	C16—C15—C20	117.7 (5)
H35—C3A—H36	109.5	C16—C15—Si1	123.3 (4)
C1—C4A—H44	109.5	C20—C15—Si1	118.9 (4)
C1—C4A—H45	109.5	C17—C16—C15	120.7 (6)
H44—C4A—H45	109.5	C17—C16—H161	119.6
C1—C4A—H46	109.5	C15—C16—H161	119.6
H44—C4A—H46	109.5	C18—C17—C16	120.4 (6)
H45—C4A—H46	109.5	C18—C17—H171	119.8
N2—C5—C6	110.7 (5)	C16—C17—H171	119.8
N2—C5—C6A	109.6 (19)	C19—C18—C17	119.8 (5)
N2—C5—C8	108.6 (4)	C19—C18—H181	120.1
C6—C5—C8	112.7 (7)	C17—C18—H181	120.1
N2—C5—C7	108.4 (4)	C18—C19—C20	119.6 (5)
C6—C5—C7	109.8 (6)	C18—C19—H191	120.2
C8—C5—C7	106.4 (6)	C20—C19—H191	120.2
N2—C5—C8A	104 (2)	C19—C20—C15	121.6 (5)
C6A—C5—C8A	130 (4)	C19—C20—H201	119.2
N2—C5—C7A	104.2 (19)	C15—C20—H201	119.2
C6A—C5—C7A	105 (4)		
			
N2—P1—N1—C1	−174.3 (4)	P1—N2—C5—C8	144.0 (5)
Cl1—P1—N1—C1	−72.2 (4)	Si1—N2—C5—C8	−24.2 (8)
N2—P1—N1—Si1	1.9 (2)	P1—N2—C5—C7	−100.8 (6)
Cl1—P1—N1—Si1	104.00 (16)	Si1—N2—C5—C7	91.0 (6)
N2—Si1—N1—C1	173.9 (4)	P1—N2—C5—C8A	−13 (3)
C9—Si1—N1—C1	−75.0 (5)	Si1—N2—C5—C8A	179 (3)
C15—Si1—N1—C1	57.8 (5)	P1—N2—C5—C7A	92 (3)
N2—Si1—N1—P1	−1.8 (2)	Si1—N2—C5—C7A	−76 (3)
C9—Si1—N1—P1	109.3 (2)	N1—Si1—C9—C14	157.3 (4)
C15—Si1—N1—P1	−118.0 (2)	N2—Si1—C9—C14	−111.2 (4)
N1—P1—N2—C5	−173.6 (4)	C15—Si1—C9—C14	23.1 (5)
Cl1—P1—N2—C5	82.8 (4)	N1—Si1—C9—C10	−29.7 (5)
N1—P1—N2—Si1	−1.9 (2)	N2—Si1—C9—C10	61.8 (4)
Cl1—P1—N2—Si1	−105.51 (15)	C15—Si1—C9—C10	−163.9 (4)
N1—Si1—N2—C5	172.4 (5)	C14—C9—C10—C11	−2.0 (8)
C9—Si1—N2—C5	59.0 (5)	Si1—C9—C10—C11	−175.4 (4)
C15—Si1—N2—C5	−73.0 (5)	C9—C10—C11—C12	0.7 (9)
N1—Si1—N2—P1	1.8 (2)	C10—C11—C12—C13	0.4 (10)
C9—Si1—N2—P1	−111.6 (2)	C11—C12—C13—C14	−0.2 (10)
C15—Si1—N2—P1	116.4 (2)	C10—C9—C14—C13	2.2 (8)
P1—N1—C1—C3A	24.1 (17)	Si1—C9—C14—C13	175.3 (4)
Si1—N1—C1—C3A	−150.6 (16)	C12—C13—C14—C9	−1.1 (9)
P1—N1—C1—C3	−145.9 (6)	N1—Si1—C15—C16	−107.9 (4)
Si1—N1—C1—C3	39.5 (8)	N2—Si1—C15—C16	158.0 (4)
P1—N1—C1—C4	−19.6 (7)	C9—Si1—C15—C16	25.9 (5)
Si1—N1—C1—C4	165.8 (5)	N1—Si1—C15—C20	69.6 (4)
P1—N1—C1—C4A	155.6 (12)	N2—Si1—C15—C20	−24.4 (4)
Si1—N1—C1—C4A	−19.0 (13)	C9—Si1—C15—C20	−156.5 (4)
P1—N1—C1—C2	97.0 (5)	C20—C15—C16—C17	2.9 (8)
Si1—N1—C1—C2	−77.7 (6)	Si1—C15—C16—C17	−179.5 (5)
P1—N1—C1—C2A	−93.8 (12)	C15—C16—C17—C18	−1.3 (9)
Si1—N1—C1—C2A	91.6 (12)	C16—C17—C18—C19	−1.0 (10)
P1—N2—C5—C6	19.7 (8)	C17—C18—C19—C20	1.6 (9)
Si1—N2—C5—C6	−148.5 (7)	C18—C19—C20—C15	0.0 (9)
P1—N2—C5—C6A	−156 (4)	C16—C15—C20—C19	−2.3 (7)
Si1—N2—C5—C6A	36 (4)	Si1—C15—C20—C19	−180.0 (4)
